# Philadelphia Hospital

**Published:** 1839-02-23

**Authors:** 


### PHILADELPHIA HOSPITAL.

DISEASES OF THE EAR.—(CONTINUED.)

*Saturday, February 9th.*—At 10 o'clock, Dr. Horner commenced:

Gentlemen,—In continuing the subject of diseases of the ear, I propose to-day to review briefly the various affections to which the organ of hearing is liable, and to enumerate the particular symptoms which characterize each form of aural disease, with an abstract of the treatment most generally approved in each case. These I shall illustrate, by introduciug such specimens as the patients of the house may present. Following the classification mentioned at our last meeting, I shall commence with the diseases of the external ear, and first of *Erysipelatous Inflammation of the Auricle.*

We have here all the ordinary appearances of erysipelatous inflammation in other parts. The auricle becomes swollen, red, tense, shining, hard, and of a dark brown colour. The sensibility of the part is morbidly increased, and this frequently remains some time after a subsidence of the other appearances. Transparent vesicles, filled with serum, form, which end in yellow purulent points, or in scabs. Constitutional symptoms are developed, with gastric irritation and a foul, loaded tongue, and some febrile movement. These symptoms last for several days, and then abate. This disease is frequently a concomitant of erysipelas of the face, and its causes are exposure to the sun, stings of insects, wounds, applications of an irritating description, &c. The general and local treatment is the same as that of erysipelas of the face. I should think it worth while to try the application of a solution of the nitrate of silver, or a mixture of wheat flour and nitrate of silver to dust the part with.

*Scirrhous degeneration of the auricle*, and of the *meatus externus* resembles, in its incipient stage, erysipelas of the same parts, and is principally distinguished from it by its protracted duration. The ear becomes thin, nodulated, and misshapen. The pain is urgent, burning, and continued. It is rarely attended with fever, and ends in ichorous ulcerations, situated in the cutis vera. In the treatment, attention must be paid to cleansing the part repeatedly, and we must depend on alteratives, purging, and counter-irritation. The sulphur baths and Zittman's decoction will be found of service, as well as the use of the tartar emetic ointment, on the neck, below the mastoid process.

*Furuncle of the auricle* resembles a common boil, and is a hard circumscribed tumour, attended with severe pricking pains, redness and heat, ending in a purulent focus, and involving the cellular texture. Its seat is generally near the meatus, or in the scapha, or the cavitas innominata. Emollient poultices are to be applied till suppuration is established, and then the boil is to be freely opened. Should the disease be extensive and severe, a proportional degree of antiphlogistic treatment must be observed.

*Erysipelatous inflammation* attacks, also, the meatus. This is accompanied by pricking, burning, itching sensations, great sensitiveness to the touch, delusive noises, confusion of head, increase of temperature, impaired facility of hearing, the ear feeling full and stopped. The ceruminous secretion becomes increased and altered, and is of a brown colour, mixed with scales of cuticle, and adhering to the meatus. Blood frequently is discharged from the meatus. The developement of this affection is generally sudden, though sometimes slow. In its treatment, we must have frequent recourse to injections of tepid water, to remove the accumulated ear-wax, which irritates the passage and augments the disease. An injection, consisting of a grain of the sugar of lead in an ounce of water, will also be found of service. When ulceration has commenced, the part must be brushed with tincture of myrrh or opium. The application of a solution of the nitrate of silver I would, also, recommend, as well as a mixture of the caustic with flour. If it be obstinate and tedious, counter-irritation beneath the mastoid process will, also, be advantageous.

The ear is subject to an affection of its glandular structure, commonly known as *Catarrhal inflammation of the Meatus Auditorius externus.* In this affection the meatus becomes swollen, its calibre is diminished, and sometimes it is entirely closed. The texture is variable, sometimes being firm and sometimes spongy, with serous or purulent vesicles. It is red, hot, and a burning, itching pain extends over the whole ear, mastoid process, and parotid gland, and it is particularly sensitive to the touch. A serous, muco-purulent, or bloody secretion, of an ammoniacal odour, is discharged; the auricle sometimes is involved. This disease is apt to be followed by granulations, which are of two kinds,—vascular, spongy, sensitive; or cartilaginous and insensate. It lasts for weeks, and, indeed, years, though, according to Kramer, it never penetrates to the bone, or induces true inflammation, or destroys the membrana tympani, although the latter has an inflammatory redness. It is produced by cold, irritating substances, foreign bodies in the meatus, and cutaneous affections. In the Children's Asylum, connected with this institution, the inmates are very liable to purulent ophthalmia, and have very frequently this as an attendant, as you will presently see. The cuticular lining of the meatus and of the membrana tympani is most frequently detached, by the disease lasting a few weeks. In the early stages, depletory treatment and antiphlogistic measures are to be employed. The passage is to be kept clean by frequent aqueous injections, which are to be warm or cold, according to the sensations of the patient. Antimonials may be administered internally, and if there be much plethora, active purging several times a week, with spare diet. In a more advanced stage, a solution of the acetate of lead, (ten grains to an ounce of water,) or of the sulphate of copper or zinc, or of the nitrate of silver may be employed. The sugar of lead water will be found best suited for correcting the ammoniacal odour of the discharge. Kreosote, pyroligneous acid, the chlorides of lime and soda, have, also, been recommended. Tartar emetic pustulation on the neck will frequently be serviceable.

This disease is commonly designated *otorrhea*, in consequence of the discharge from the ear, which may sometimes be an inconsiderable quantity of serum, scarcely attracting attention, and in other cases, again, is so copious as to soil several cloths daily with a sero-purulent fluid.

When polypous granulations arise, all emollient and oleo-mucilaginous applications are to be repudiated. Kramer prefers the use of a sponge compress. If the polypi be very large, they may be removed by the ligature, or by excision, or by touching them with the solid lunar caustic. When in a chronic indurated state and near the membrana tympani, they are cured with much difficulty.

Insects may be removed, by filling the meatus with olive oil, and foreign bodies are to be extracted by the forceps or probe.

[Several cases of this disease, in children, were introduced and commented on.]

*Inflammation of the cellular tissue* of the meatus, or *phlegmonous inflammation*, is marked by swelling and tension. There is an elevated spot, the size of a pea, generally in front, and resembling a common boil. Sensitiveness is extreme, the pain severe, and there is considerable dullness of hearing, with delusive noises. The patient has fever, and frequently hemicrania. The little tumour, at from three to seven days, breaks, and discharges a bloody pus. The invariable termination of this disease is suppuration. The remedies, of course, are such as will hasten this process—emollient applications, &c. Purging is sometimes necessary, and the patient must be kept on low diet.

*Inflammation of the periosteum of the meatus auditorius* is characterized by a circumscribed swelling deep in the canal, unaccompanied with much pain; which opens, and discharges a thin, fœtid, filthy pus. On exploring the auditory passage with a probe, an exposed and carious bony surface is detected. The progress of this affection is slow; it arises sometimes from scrofula, or the metastasis of exanthematous diseases, as measles, scarlatina, and small-pox. Kramer regards the prognosis as unfavourable, when such are its causes. The topical treatment consists in ablutions of tepid water, and an avoidance of any irritating applications. If it be the result of constitutional disorders, our attention must be directed to the treatment of these.

If obliteration of the meatus occurs, it must be opened with a trocar. Kramer states that none of his patients had sufficient patience to await the period of cure, so that he does not report any complete cases.

The next disease of the ear to which I shall call your attention, is *Acute Inflammation of the Membrana Tympani.* The patient suffers from acute, tearing, dragging pains at the bottom of the meatus, and experiences the sensation of an insect in the canal. The hearing is imperfect and painful, with constant susurrus or tinnitus. On examining the membrane of the tympanum, it will be found swollen, protuberant, and of a blood-red color. General symptoms, as fever, restlessness, &c., commonly attend this complaint. The discharge from the ear is generally watery, purulent, or mixed. It terminates either in ulceration or thickening and opacity of the membrana tympani, and sometimes in a cartilaginous degeneration. Cold and acrid applications are its common causes, and, according to the observations of Mr. Kramer, never an accumulation of ear-wax. Its duration is from a few days to some months. Antiphlogistic treatment and regimen must be employed, as leeches, saline purges, &c. The best local application during the acute stage, is warm, sweet wine. After the acute inflammatory stage has passed, injections of a solution of the acetate of lead are of service, but care must be taken not to use them too soon, or they will favour the thickening of the membrane. Counter-irritation, with the tartar emetic ointment, will be found useful. Mr. Kramer believes that a hole in the membrane of the tympanum is always a consequence of preceding inflammation, and that the facility of hearing is always impaired by it. He rejects entirely the idea of relaxation or preternatural tension of the tympanal membrane.

The membrana tympani should only be perforated when a cartilaginous degeneration is fully ascertained. The success of this operation depends on the permanence of the opening, and a small, circular piece of the membrane should be removed by a gentle rotatory motion of Himley's punch. Should a purulent discharge follow the puncture, we may conclude that disease of the middle ear exists, and no benefit will accrue.

The membrane of the tympanum is also liable to *chronic inflammation*, and when in this state, on examination we find it thickened, uneven, swollen, opaque, red, and sometimes ligamentous or cartilaginous. It is sometimes ulcerated, and sometimes granulations spring up from it. The sensibility varies,—being, in some cases, not beyond the natural standard, and in others, considerably exalted. There is tinnitus, with dulness of hearing, and a serous or muco-purulent discharge, which is unsettled in quantity, and sometimes concretes in crusts. The ceruminous secretion is suspended. The disease is insidious in its onset, and may last for years and even during life. The prognosis is, in general, unfavourable, although amelioration does sometimes take place. The treatment is pretty nearly the same as that previously detailed. The fœtor of the discharge may be corrected by the acetate of lead, in solution. This affection commonly terminates in perforation of the membrana tympani.

I shall now proceed to the consideration of the diseases of the middle ear, under which head, I shall treat of the affections of the cavity of the tympanum and Eustachian tube, offering to you an abstract of the symptoms and general treatment; my time not allowing me to enter more fully into the subject at this time.

In inflammation of the *mucous membrane* of the middle ear with accumulation of mucus, there is a fulness and weight in the part, accompanied with little or no pain, and some dulness of hearing, as if a veil were thrown over the ear. There are sometimes tinnitus and a crackling sensation in the ear, although both may be wanting. Audition, in some cases, is improved by warm weather, exercise or clearing the throat, and diminished by cold, dampness, catarrh, the depressing passions; though in others, again, these appear to exercise no influence. Inflammation of the mucous membrane of the tympanal cavity is frequently complicated with chronic inflammation of the throat, palate and tonsils, and is accompanied with a stuffing in the head, an abundant secretion from the mouth and nose, and sometimes by a relaxed pendulous uvula. It is a disease commonly of early life. The general treatment found most beneficial is a dry strong diet, the use of Zittman's decoction, free purging, iodine. The local treatment consists in leeches, cold ablutions to neck, face and throat, to be followed by dry frictions, and counter-irritation. The introduction of a current of air, or the injection of water, with a little common salt dissolved in it, is a favourite remedy; the Eustachian tube being used for the purpose. The sign of a free passage and unobstructed state of the Eustachian tube, is, that the air, on applying your ear to the patient's, is heard to pass into the tympanum, by an uniform, broad, rushing current. It appears to strike the membrana tympani, and issue from the meatus of the patient. When the tube is obstructed with mucus, there is a gurgling, interrupted sound, which is, also, fine, shrill, and somewhat like a squeak. If the dulness of hearing depend simply on an obstruction of mucus in the tympanum and the tube, there is always an immediate improvement after the air-douche, though the patient may relapse again in a short time, until the pathological condition of the mucous membrane is removed. The complete cure of the mucous membrane is ascertained by the enduring improvement in audition, and also by the broad, free sound having taken the place uniformly of the gurgling one. If, after four trials with the air-douche, no sound is heard in the tympannm, it may be considered that the Eustachian tube is strictured or even obliterated. If strictured, it must be dilated with a catgut bougie; if obliterated, treatment will be unavailing. The graduated sizes of the catgut, are harp E, guitar E, violin E, guitar A, violin A, Harp A.

*Inflammation of the Eustachian tube*, with stricture, is characterized by great dulness of hearing and ideal noises, though the latter are not invariably present. Chronic inflammation and thickening of the fauces and palate, always attend this affection. It is impossible to inject air or water, and on attempting to pass the catgut bougie through the tube, on reaching the strictured point, it is arrested. The prognosis is unfavourable.

The same symptoms as those just detailed exist in inflammation with tumefaction of the Eustachian tube, except that the bougie can be passed into the tympanal cavity. This disease is attended with a collection of mucus in the tympanum. The prognosis is, likewise, rather unfavourable. The treatment, in both disorders, consists in leeches, purges, emetics and iodine, light diet and the passage of catgut bougies. A revulsion with tartar emetic ointment is also indicated.

Obliteration of the Eustachian tube is known by excessive dulness of hearing, and the impossibility of passing the bougie. It is considered incurable by Kramer, who rejects absolutely the idea of Saissy, to open it with a stilet; and of Perrin, to cauterize it through with Perrin's instrument.

The next affection of the ear I shall speak of is *Acute Inflammation of the Cellular Tissue* and of the *Periosteum of the Tympanum*, which is characterized by severe burning pain, extending to the pharynx, and to the side of the head. The audition is either dull or very much excited, confused, and attended with ideal sounds. The membrana tympani is inflamed and sensitive. The Eustachian tube is also sensitive to the touch. Pain is experienced on any motion of the jaw, or sneezing, and invades, finally, the entire side of the head. The eye of the same side is very red, weeping, and sensitive to light. No alteration occurs in the meatus commonly. There are redness and tumefaction over the mastoid, which finally end in suppuration. The epiphenomena are fever, with alternations of chilliness and heat, which are severe in the evening and remit in the morning. In the worst cases, the pain and the constitutional disturbance are so great, that there is a raging delirium; hard, quick pulse, great heat of skin, urgent thirst,constipation, high-colored urine, vertigo, loss of appetite, and vomiting. This state lasts for a few days, when the patient suddenly dies, apoplectic; or under the most exasperated symptoms of phrenitis. It is particularly insidious in children, because, from their want of definite words, the disease advances into the stage when the cerebral symptoms are most prominent, without an affection of the ear being suspected. Some intimation of its implication is commonly given by their carrying their hand frequently to the part. Under ordinary circumstances, this disease may last from a few days to months. The symptoms are exasperated from day to day, until a purulent, bloody fluid finally bursts through the meatus externus, bringing away with it fragments of the temporal bone and the ossicula auditus. A livid red colour over the mastoid process is not unfrequently a sure guide to this affection, and where suppuration is established in it, a probe can be carried down to the bone.

The same disease may exist in a chronic form, and the symptoms are nearly the same. On the dissection of cases of both acute and chronic inflammation of these parts, pus is found in the cavity of the tympanum, in the labyrinth, and in the cells of the mastoid process. The dura mater, in the region of the temporal bone is found detached, discoloured and suppurating, and the arachnoid membrane is sometimes involved. The most energetic antiphlogistic treatment is demanded. I shall here read you a case of this disease which terminated favourably, and which I attended in consultation with Dr. Pancoast. He having allowed me the use of his journal of the case, I have, through his kindness, formed from it this abstract.

*May* 23*d*, 1828.—Eliza McElroy, aged 20, a domestic, of sanguineo-nervous temperament, has, during her whole life, been subject to earache, frequently repeated, and attended with discharges from the meatus. Several sisters have been afflicted likewise.

For the last six months every exposure to cold, though trifling, has produced severe pain in the right ear, and a less degree of it in the left. She now has fever, and general uneasiness, in addition to the ordinary sensibility of her ear. She was relieved by bleeding, a cathartic, and avoiding exposure.

*May* 26*th*, 1828.—Slight and incessant pain in the right ear. The next day she had pain in both ears—that of the right being excessive, and attended with a small discharge of bloody serum, and some fever. Up to the 30th, there was no material change. The bloody serum then came away in large quantity, and brought with it small fragments of bone. A slight discharge from left ear.

The next day the symptoms continuing, to them were added a convulsive drawing of the right eye upwards, and a vomiting of every thing. The day after, there was soreness over the mastoid process of the right side. Hearing perfect in each ear, and the left ceased to be troublesome.

Up to the 5th of June, some improvement,—a hole, which had been seen on the 31st ult. in the membrana tympani, was now much enlarged.

*June* 12*th*, 13*th*.—A decided relapse; increase of pain, of discharge, and of fever; discharges of bone; tenderness behind right ear. On the 18th she had delirium, which continued with some intermission to the 24th. On the 25th delirium returned, with stridulous breathing, and occasional spasms; throat inflamed.

*June* 26*th*.—Short but violent spasms of the glottis, and of frequent recurrence. Frequent discharges of sero-sanguineous fluid into the pharynx, each preceded by much difficulty in breathing: the next day it was discharged through both mouth and nostrils, and rendered the breath fœtid.

In the interval to July 6th, she had discharged several large pieces of bone, both through the pharynx and the meatus externus. A loose bone was then felt by the patient to move about considerably, as the posture of the head changed. The last evening a total blindness of both eyes supervened—the first to be attacked, and the last to recover, being the right. This attack was preceded by intense pain, partial loss of hearing in left ear, that of the right remaining perfect.

In the afternoon of the 7th of July, agonizing pain in the head, like the passing of a waggon wheel over it; it was quickly followed by perfect blindness on both sides, with partial loss of smell and taste. The vision returned in a few minutes, but she became perfectly deaf, with excessive palpitations of the heart, dyspnœa, and epigastric uneasiness, excessive itching in skin of upper part of the face and in the nostrils, clammy and itching sensation in lining of mouth—coldness felt in the throat.

The next day she was more comfortable, with perfect hearing in both ears,—with vision, taste, and smell, not much impaired. The symptoms continued, with alternations for the better or worse, till the 10th, when she had several severe and universal spasms. They were preceded by severe pain, with heat in the occipital region. From this period to the 18th, there were various attacks of spasm, partial or universal, abolition and restoration of the senses, inability to open the mouth, pain, dyspnœa, prickling sensation in her right arm and leg in the course of the nerves, want of motion in the limbs.

The patient had convalesced so far by the 6th day of August, as to be able to go into the country. I saw her twice during her illness, in consultation with Dr. Pancoast; and Dr. Jackson, during an absence of Dr. P. from the city, had her under charge from June 28th to July 4th.

I have omitted Dr. P.'s details of treatment, but they were very judicious, being of an active, antiphlogistic kind, attended occasionally with blisters and sinapisms.

*February* 4*th*, 1839.—She is now in good health, and hears on both sides. On weighing the fragments of bone, they amount to ʒij. in the dried state, and number thirty-six, great and small. They vary from the size of a grain of wheat, to that of the end of the finger, and are principally of the cellular structure—some few of the pieces being smooth on the surface, exhibit thereby the plate next to the dura mater, and portions of the foramina of the contiguous part of the skull. They seem to be from the petrous, mastoid, and occipital bone, though the fragments are too irregular to determine the precise portions.

In this box I exhibit to you the collection of bony fragments, as preserved by Dr. Pancoast, it is almost incredible that such destruction should have occurred, and the patient survive it. No one, to have seen her in one of her paroxysms of frantic delirium, would have anticipated such a result.

I shall now pass to the consideration of the diseases which assail the internal ear. A disease of this part can only be rigidly inferred, when we have ascertained by the speculum and lantern, that there is no affection of the meatus externus, or membrana tympani; likewise by insufflation and the catgut bougie, that the Eustachian tube and tympanum are free from disease. An impaired function of the labyrinth appears under two forms. 1. An augmented irritability—erythismus. 2. Diminished irritability—torpor. Tinnitus is an essential sign, being present in the first, and absent in the last.

In *Erethitic nervous deafness*, there are constant ideal noises, as buzzing, the sound of wind, rain on trees or houses, roaring of the sea, afterwards becoming like the chirping of birds, whistling, or a kettle of boiling water. These delusive sensations are increased by violent exertions, and by excitements. The dulness of audition is increased by bad weather, and finally it becomes almost obliterated. Hearing is facilitated for the time, by the rattling of carts, or any heavy jarring noises, as a peal of bells, or the beating of a drum. It is rendered more imperfect by brazen instruments of music. A loud shrill voice is insupportable; the opposite is sometimes pleasant. The disease having advanced to its acme, perfect deafness then follows, and the tinnitus finally ceases. Acuteness of vision in distinguishing the motion of the lips of a speaker, is sometimes mistaken by the patient for improved hearing. By insufflation with the air-douche, the hearing is made worse, the head confused, and the ear feels stuffed. A gradual diminution in the cerumen occurs, though this is not an invariable occurrence. The meatus is dry, scaly, and insensible. The auricle is in the same state. The treatment is the introduction of the vapour of acetic ether, very gradually into the middle ear through the Eustachian tube, according to the method described in the last lecture, and the effect on the hearing is to be cautiously observed and noted.

I shall conclude with a few words on the *Torpid form of nervous deafness.* Here there is a gradual and total privation of audition, with a suspension of the sensibility of the meatus and auricle, and of the ceruminous secretion. As I before mentioned, there is no tinnitus. The membrana tympani is opake, like white paper. The Eustachian tube and meatus externus are generally open, but if they be obstructed, no relief can be anticipated. When a strong watch cannot be heard, the prognosis is unfavourable. The only treatment which is entitled to any confidence, is the introduction of the vapour of strong warm acetic ether, as in the erithitic form, into the Eustachian tube.

The treatment of nervous deafness, should be continued for at least three months, and the longer the better. Tinnitus is no proof of nervous deafness, as it attends all aural diseases, any more than it is indicative of sanguineous congestion in the head.

The case which I shall now narrate, shows the improvement in the power of hearing, under the influence of a stream of ethereal vapour, continued almost daily for some time. I first used sulphuric ether, and then acetic ether. My condenser, holding a quart, was filled with a second atmosphere of air, impregnated with the ether, by passing the air through the ether, and the stream of medicated air was then let into the Eustachian tube. Each ear received generally at a sitting, two douches, prepared in that way, or half a gallon of medicated air.

*Nervous Deafness from Infancy, attended with some obstruction of the Eustachian Tube from Mucus.*

Mr. C., æt. 21. June, 1838.

*9th.*—Hearing distance with my watch on right side, 18 inches; ditto, left side, 12 ditto.

*11th.*—Hearing distance with my watch on right side, 20 inches; ditto, left side, 17 ditto. On each side hears two inches more after injection of vapour of sulph. æth. Clear, hot day.

*12th.*—Clear, hot day; treatment repeated. Hears 21 inches on right side, 23 on left side, before injection; considerable tumefaction in region of parotid gland, on left side, from emphysema.

*13th.*—Did nothing.

*14th.*—Tumefaction diminished. Hearing distance on both sides, 2 feet 6 inches; distended with 1 quart on left side, 2 quarts on right, of vapour acet. æther; hearing distance increased on right side, to 3 feet; on left, to 2 feet 9 inches.

*15th.*—Audition same, repeated process.

*16th.*—Ditto, ditto.

*17th.*—No injection; dose of Epsom salts.

*18th.*—Hearing distance on right side, 3 feet; ditto, on left side, 2 feet 9 inches; at end of douche, 3 feet 3 inches on right side; ditto, 3 feet on left side.

*19th.*—Hearing distance on right side, 3 feet 6 inches; ditto, on left side, 3 feet 3 inches. After the ethereal air-douche, hearing increased on right side, to 3 feet 9 inches; ditto, on left side, 3 feet 6 inches.

*20th.*—Ditto, ditto.

*21st.*—Ditto, right side, 4 feet; ditto, left side, 3 feet 6 inches. After douche, ditto, right side, 4 feet 6 inches; ditto, left side, 4 feet.

*22d.*—Ditto, as after douche of yesterday.

*23d.*—Ditto, as before douche. After ditto, on right side, 5 feet; ditto, on left side, 4 feet 3 inches.

*24th.*—Nothing done.

*25th.*—Before douche, hearing as last report. Gave douche in each of fumes of acet. æther. Hearing distance, on each side, near 6 feet.

*26th.*—Before douche, hearing distance in each ear, 7 feet.

*27th.*—So much noise in street, no test would answer.

*28th.*—Before douche, 7 feet on each side.

*29th.*—Ditto, 9 feet ditto.

*July 1st.*—Ditto, streets very noiseless; 13 do.

*July 6th.*—Repeated douche of acetic æther, each day, till present, inclusive. Hearing distance, on right side, 13 feet; ditto, on left side, 14 feet.

*Feb. 17th*, 1839.—By a letter received this month from the gentleman in question, it appears that he hears as well as when the treatment was suspended. In his communication, however, he expresses himself uncertainly in regard to his power of audition generally, and seems anxious to renew the treatment. Cold, he says, affects his hearing very much.

[During the lecture, several patients were introduced, labouring under various diseases of the ear, and their cases explained and operated on; but we have preferred presenting an uninterrupted line of the lecture, believing that it would, in this way, be more useful and acceptable.]

